# The effectiveness of the use of a technology toolkit on activities and mother-child interactions: children with complex care needs

**DOI:** 10.1080/17483107.2023.2293870

**Published:** 2023-12-19

**Authors:** G. F. Bos, E. van Wingerden, P. S. Sterkenburg

**Affiliations:** a Department of Care Ethics, University of Humanistic Studies, Utrecht, Netherlands; b Clinical Child and Family Studies and Amsterdam Public Health Research Institute, Vrije Universiteit, Amsterdam, Netherlands; c Department of Assessment and Treatment, Bartiméus, Netherlands

**Keywords:** Mother-child interaction, children, intellectual disability, profound disabilities, assistive technology, participant observation

## Abstract

**Purpose:**

Meaningful interactions with significant others are crucial for children’s well-being, including those with severe to profound intellectual and multiple disabilities. This study aimed to gain more insight into the potential of an ICT-Toolkit in enhancing or contributing to the interaction between parents and their children with complex care needs.

**Methods:**

A multiple case study with an AB experimental design was conducted. Four mother-child dyads were observed during eight two-and-a-half-hour home visits. Children between 6 and 16 years with severe to profound intellectual and multiple disabilities were included. A participatory research observation approach was used.

**Results:**

Using the ICT-Toolkit compared to the baseline phase stimulated a decrease in the repetitive activities of two of the four dyads. Overall, the activity repertoire of the children and the length of time being engaged in individual activities increased, and the activities together increased in three of the dyads. There were more turn-taking interactions; the children took more initiative to engage their mother in their activity, and the children exhibited more affection when engaging with the ICT-Toolkit items. Three mothers followed their children more in their play, and all mothers were more verbally and physically active in instructing and/or motivating their children.

**Discussion:**

All mothers noticed their child’s “new” behaviour when interacting with an ICT-Toolkit item. The mothers followed the child’s lead when engaged with the ICT-Toolkit. The ICT-Toolkit’s added effect may be due to the predictability, uncomplicated and highly perceivable stimuli it provides with minimal effort and maximum effect.

## IMPLICATION OF REHABILITATION

- A technology toolkit contributes to activities and mother-child interactions for children with intensive care needs.
- The ICT-Toolkit contributes to more turn-taking interactions and more initiative taking by the children to engage their mother in their activity.
- Children exhibited more affection towards their mothers when engaging with the ICT-Toolkit items.
- The participation in this study led mothers of children with intensive care needs to mentalise about the needs of their child and how their behaviour may influence the behaviour of their child.
- The ICT-Toolkit’s added effect may be due to the predictability, uncomplicated and highly perceivable stimuli it provides with minimal effort and maximum effect.

## Introduction

### Background

Playful social interaction with significant others is crucial for children’s social and cognitive development, including those with severe to profound intellectual and multiple disabilities, henceforth referred to as children with complex care needs. Similarly, for adults with and without complex care needs, sharing meaningful moments and having fun together provides a foundation for social interaction [1]. However, engaging in mutually fulfilling interactions can be challenging for people with and without complex care needs. Challenges for parents and caregivers include the difficulty for parents and caregivers to understand the needs of children with disabilities [2,3] as well as the striking asymmetry in physical, cognitive and communicative abilities and preferences of children with complex care needs during the interaction [4].

When living at their family home, the social network addressing the functional and social interaction of children with complex care needs is often restricted to direct family members and professional caregivers [5, 6]. It can be challenging for parents to involve their child with complex care needs in meaningful family activities that all family members can enjoy. Consequently, in everyday life, the activities of children with complex care needs tend to be less diverse than those of typically developing children, and they show less engagement during those activities [7,8].

### Interaction

Interaction is generally considered meaningful when there is an (in-depth) social and engaging component next to, or instead of, a functional component. A specific partner attunement style is required to engage in meaningful interaction during joint activities with persons with disabilities and/or complex care needs [2,9,10]. Factors contributing to this attunement style are sensitive responsiveness, joint attention, co-regulation and an emotional component [11]. Also [10] noticed that meaningful interaction is influenced by the physical context, which can augment tangible conversation topics and structures for shared attention.

In the case of children with complex care needs, the attunement of the interaction partner means being sensitive and responsive and able to adapt communication strategies to the abilities and preferences of the person with complex care needs [9,12,13]. Furthermore, the physical setting and suitable stimuli are essential for meaningful interaction [10]. Parents, for instance, must be flexible and receptive enough to attune to their child’s communication style. Attempts of reciprocity by children with complex care needs are often distorted and subtle. It is, therefore, challenging to detect the child’s attempts at intentional communication [14] and even harder to interpret unintentional communication. As a result, navigating the communicative possibilities for joyous, meaningful interactions are challenging [1,6,15].

Parents often develop and foster familiar or routine interactions with their children with complex care needs strongly related to their daily practical needs and caregiving [16,17]. On the one hand, this may help both parties avoid frustration and communication breakdowns, but on the other hand, it may hinder further interactional development regarding meaningful interaction.

Since the development of children with complex care needs is delayed compared to children without complex care needs [18], and their everyday life often consists of fixed structures, repetitive routines and little variation [7], both the context and the content of their interactions with others tend to be predictable. This context/setting provides a sense of safety, which is vital for people with complex care needs. However, the routine’s predictability and repetitive nature do not encourage engagement in meaningful interactions between family members with and without complex care needs.

### The use of technology in the homes of children with complex care needs

Many studies have demonstrated that persons with complex care needs can use assistive technology and how this can improve their participation and active engagement in leisure activities and social interactions [19,20]. A wide range of assistive technology aims to improve individuals’ daily functioning. Both low-tech devices, such as micro switches, and high-tech devices, like text-to-speech computer software, may be suitable for children with complex care needs [21]. Since these products can provide a basis for interaction [22,23], assistive technology can contribute to the interaction between parents and their children with complex care needs. Indeed, some products enable joint activity between parent and child, whereas others can support communication [24]. For instance, speech buttons or speech computers allow the child to draw others’ attention, initiate interaction, express themselves more clearly, and even convey more complex signals [25,26]. In addition, microswitches combined with power switches can offer a child control over certain household appliances or toys. As such, technology can give children with complex care needs more control and the possibility to take more initiative in their interaction with their parents [20,22,27].

However, persons with complex care needs depend on their caregivers to access these devices. Caregivers are required to make materials available to the person with complex care needs and actively use them. A recent questionnaire among 195 *professional* caregivers of persons with complex care needs [28] showed that the knowledge and experience of professional caregivers with technology were related to their motivation to use the technology available for their clients. Participants with more knowledge and experience were more likely to overcome practical barriers such as planning or the challenges or complexity of the child’s (dis)abilities and preferences. Less than half of the participants in this study estimated that they had enough knowledge or experience to use assistive technology for persons with complex care needs.

Until now, it is unclear whether parents (and other *non-professional* caregivers) are aware of the available assistive technology, how to access it, or can sufficiently estimate what would be the most beneficial for their interactions. Research indicates that parents might need support in finding suitable products regarding the abilities and preferences of their child in their personal situation [29].

### The present study

This study provides assistive technology to parents of children with complex care needs. Four families were provided with knowledge and the opportunity to gain experience with a basic set of technological products suitable for their children, the *ICT-Toolkit* [30]. This paper examines the influence of using readily available assistive technology, an ICT-toolkit developed for parents and caregivers of persons with complex care needs, on the interactions between dyads. Specifically, we explored their involvement, mutual attentiveness, and activity frequency and variation.

The study’s objectives were to explore: (1) How the ICT-Toolkit influenced the activities undertaken by parents and children? (2) How parents and children applied the ICT-Toolkit to facilitate meaningful interaction? and (3) Which elements of the ICT-Toolkit were most attractive to the parents (concerning interaction and shared activities with their child)? It was expected that applying the ICT-toolkit this would result in (temporary) changes in the predictable context of parent and child, possibly resulting in better (mutual) attunement because the child could engage in new ways of expression and participation [10].

## Method

### Design

A multiple case study was performed with an AB experimental design, including four families with a child with complex care needs, to gain more insight into the potential of the ICT-Toolkit (see Table 1). First, a short interview (condition P) was conducted, focussing on the parents’ expectations. Second, four 2.5-h observations for each participant were conducted during the baseline, condition A. Third, the families received the ICT-Toolkit. Fourth, during the intervention phase, condition B, again, for each participant, there were four 2.5-h observations. Fifth, an evaluative interview in each family concluded the intervention phase. During the home visits, a participatory research observation approach was used to document any developments in the activities and interaction between parent and child.

**Table 1.** Research design overview.

Participant & Interview	Baseline (4 weeks)				Intervention (4 weeks –> 8 weeks)				Interview
1 I1	A1	A2	A3	A4	B1	B2	B3	B4	I2
2 I1	A1	A2	A3	A4	B1	B2	B3	B4	I2
3 I1	A1	A2	A3	–	B1	B2	–	–	I2
4 I1	A1	A2	A3	A4	B1	B2	B3	B4	I2

Note: I = Interview; A = baseline; B = intervention; – = no data collected due to Covid restrictions.

### Participants

The main participants comprised four mother-child dyads with complex care needs. In the Netherlands, the four mothers were recruited using purposive sampling through social media messages in a large online Facebook community for caregivers of children with complex care needs. Inclusion criteria were: (a) the parent of a child with complex care needs living at home and (b) the chronological age of the child between 6 and 16 years of age. Applicants were then contacted by the coordinating researcher (EvW) and provided with extensive information about the study. Exclusion criteria were: (a) children who are seriously ill and (b) children who will have to be admitted to hospital for treatment. The native language of the parent was neither an inclusion nor an exclusion criterion.

Without exception, the four participating children attended a daycare centre multiple days a week. For the remaining time, their mother was their primary caregiver, assisted by one or more care professionals. The fathers were employed full-time and involved in (care) activities during evenings and weekends.

An interview with parents before the start of the intervention revealed that the families had not used assistive technology much because they were unaware of the available products, even though all four mothers were familiar with online video clips demonstrating assistive technology use circulating on social media. Nevertheless, they experienced obstacles to purchase a product, partly because they did not know where to buy it (there is a perceived lack of a transparent and informative platform) and partly because they did not know which product (or variant) would suit their child. The latter is even more pressing since many specially designed products are expensive. Opportunities for orientation on possibly relevant assistive products are even more limited, as their everyday family life is often very demanding and sometimes characterised by routines. In line with this, the mothers mentioned that they did not have a clear idea concerning the types of products, the purpose or the potential of the ICT-Toolkit. However, they were curious about what the ICT-Toolkit would involve and provide.

Andy (15 years) lives with his two parents and older sister. Andy has Down syndrome, a severe intellectual disability and epilepsy. Although he has motor difficulties, he can walk without support and has sufficient fine motor skills to hold and manipulate small objects. His perception seems limited to his immediate surroundings, but he has no diagnosed sensory issues. Although Andy’s mother is highly motivated to explore new ways to interact meaningfully with her son, she mentioned that she has no genuine interest in technology – unlike Andy’s father. She said she hoped that the Toolkit contained products that would enable Andy to stay focused and to practice using his hand functionally. Besides, she hoped the Toolkit would expand their opportunities for mutually fulfilling indoor activities (in case of bad weather), which she always sought.

Brian (8 years) lives with his parents and a younger brother. Brian has a severe intellectual disability. Generally, he uses a wheelchair, although when an adult fully supports him, he occasionally takes a few steps. He uses his hands to grab and point at objects but barely uses sign language and speech. Brian is easily distracted and frequently starts throwing toys or other objects when he becomes distracted. He loves listening to music and songs. His mother is keen to identify more options for prolonged solitary activities. Like Andy, Brian’s mother also expressed the hope of expanding their options for pleasant indoor activities, both shared and individual.

Colin (12 years) is one of the triplet brothers and lives with both his parents. Unlike his siblings, Colin has a profound intellectual and physical disability and epilepsy. He mostly lays down in bed since he cannot sit independently. Colin barely uses his arms and legs and does not use his hands. His epilepsy is easily triggered by sudden sounds that startle him. His mother is particularly interested in the potential of eye-tracking applications in her search for new ways to stimulate learning and create more experiences. She hoped that assistive technology could enable Colin to express what he wanted (in an action-reaction style) and to be less dependent on others to engage in activities. She reported low expectations beforehand, as Colin is profoundly disabled.

Dana (6 years) lives with her parents and younger brother. Apart from autism and a severe intellectual disability, Dana has nystagmus and epilepsy. She can sit and walk without support, although she often prefers her wheelchair. Dana has sufficient fine motor skills to manipulate smaller objects. She is particularly interested in mechanical sounds and vibrations. Her mother is an outspoken proponent of technological assistance and artificial intelligence in care activities. She expressed no specific expectations regarding Dana and the ICT-Toolkit. Instead, she said she participated in helping stimulate awareness of the use of assistive technology by people with severe and multiple disabilities. She hoped that more research would facilitate the accessibility of these devices, as she finds them promising.

### Intervention: ICT-Toolkit

In the preparation phase, four experienced advisors in the field of assistive technology for people with complex care needs were asked for their recommendations for the *content* of the ICT-Toolkit. Furthermore, the *user information* featured in the ICT-Toolkit was chosen based on the outcomes of a small survey among 18 parents and 13 professional caregivers of children with complex care needs about their primary needs for information (internal report). Consequently, the ICT-Toolkit (see Table 2) consisted of a set of (1) twelve technological products and (2) corresponding instruction videos provided on a tablet computer, (3) suggestions for using the products and (4) background information about assistive technology in general. The main product categories were (1) microswitches, (2) computer interfaces, (3) store-bought toys, (4) music devices, and (5) communication aids. The products, instruction videos, suggestions, and general information formed a basic introduction to purposefully using assistive technology and provided do’s and don’ts based on user experiences and scientific insights.

**Table 2.** Content of the ICT-Toolkit.

Background information	
Brief instructions about the study How to introduce a new product to your child Microswitches and relay boxes Augmentative and alternative communication Digital and online games and activities The power of music Finding products on the regular consumers market	
Products in the box	Information about each of the products
Smoothie microswitch PAL pad microswitch Power switch Talking tiles Story sequencer Computer interface for microswitch Eye tracker (gamers edition) A police car for microswitch Electronic dice for microswitch Talking and recording parrot An interactive ball with music and lights Vibrating Bluetooth speaker	Price category Connections, batteries, etc. Whether to combine with other devices Size Material What does it do? Which senses the product stimulates Developmental skills the product stimulates Suggestions for using or playing with the product at different cognitive or motor skill levels and for different degrees of interaction. A short movie clip demonstrating how to turn the product on and how to use it

The ICT-Toolkit contains two main sections. The first section introduces several topics about play and communication between parents and their children. The second section describes specific products representing a larger category, i.e., microswitches, computer interfaces, store-bought toys, music devices, and communication aids.

### Procedure

The Scientific and Ethical Review Board (VCWE-2019-119) of the Faculty of Behaviour and Movement Sciences, Vrije Universiteit Amsterdam, approved the research proposal. The mothers who responded to a general invitation on social media received further information and signed an informed consent form before the start of the study. Participant observation was performed by an experienced field researcher (GB), who was informed about the study’s goals but was not involved in developing the ICT-Toolkit. During the data collection process, the field researcher (GB) and coordinating researcher (EvW) kept the exchange of information about the observations to a minimum.

Data collection was split up into two rounds. The baseline and experimental phases were four weeks in the first round (autumn 2019). However, the two mothers who participated in round 1 claimed that the intervention phase of four weeks was too short to use the ICT-Toolkit to the extent they would have liked. Therefore, in the second data collection round (early spring 2020), the intervention phase was extended to eight weeks, with biweekly home observations (see Table 1). Due to the corona lockdown, the last two observations for Colin and his mother were cancelled, in addition to an appointment that had been cancelled during baseline for other reasons. Due to the lockdown, the intervention phase for Dana was postponed for three months.

After the four 2,5h baseline observations of each mother-child dyad, the coordinating researcher (EvW) provided them with the ICT-Toolkit. During this home visit, the researcher (EvW) only briefly introduced the contents of the ICT-Toolkit since all relevant information and instruction videos were available on an included tablet computer. In the second round, a printed version of these instructions was also provided based on the feedback of the first two mothers. No further instructions were given to the parents. The ICT-Toolkit remained with the family during the 4 to 8 weeks intervention phase. After the intervention phase (also four observations of 2,5 h each), the researcher (EvW) picked up the ICT-Toolkit and conducted an evaluative interview.

### Data collection

Throughout baseline and intervention phases, the field researcher (GB) observed the participants at home on eight separate occasions, 2,5 h each. Everyday family life was observed while participating in activities such as: walking the dog, having coffee, breakfast and dinner, reading a book, listening to music and grocery shopping. The documented field notes were based on direct observations as well as informal interviews about parent-child interactions and activities that were undertaken. Each home visit resulted in one observation report (29 reports).

At the end of the intervention phase, the ICT-Toolkit was collected at the family’s home. During this appointment, a semi-structured interview was conducted with the participating mothers. The researcher (EvW), who conducted these interviews, had no prior knowledge about the observation reports to remain unbiased. This interview focused on the participating mother’s experiences with the ICT-Toolkit and their participation in the study. These four interviews were recorded, transcribed, and added to the dataset (ending in 33 reports).

### Data analysis

The participating mothers were invited to correct or comment on the observation reports regarding their dyad before they were released for further analysis. Interviews were transcribed verbatim and added to the dataset. Next, thematic analysis [31] was used to identify themes inductively within the reports to work towards broader generalisations and theories [32]. In line with the research questions, the analysis focused on activities undertaken by the participating mother-and-child dyads, meaningful interaction/communication, and comments about the ICT-Toolkit. Interactions with third parties (i.e., siblings, the father, and the field researcher) were not coded.

The thematic analysis was data-driven and explorative. The first step was to become familiar with the data. Observation reports and the final interview transcripts were read, re-read, and discussed by three researchers (EvW and two independent researchers). Relevant text fragments were highlighted during this phase, and a first coding scheme was set up. In the second step, all the reports were loaded into Atlas.ti 22 (Version 22.2.0.225) [33], and the data was coded in line with the three main themes, i.e., *Activities* (sub-questions 1), *Communication* (sub-questions 2) and *About the ICT-Toolkit* (sub-questions 3). The codes indicated who made a statement or action by adding the letters “O” (parent) or “K” (child) in the code. When something was said in a text fragment about the ICT-toolkit, a code was given starting with the letter “T”; when an activity was undertaken, the code was given the letter “A”. The third step consisted of refining the themes. In discussion with the third author (PSS), the theme of *Communication* was divided into *Communication by the child* and *Communication by the moth*er. The division allowed for greater insight into the contribution of either of them. In the fourth step, all the data was coded (EvW), and a research assistant independently double-coded 30% of the data. In the fifth step, the field researcher (GB) revised and refined code groups. The consensus was met between all involved in a back-and-forth coding process, and finally, in step six, the report was written. The member check [34] did not result in any changes.

## Results

Qualitative analyses of the reports examined the influence of the ICT-toolkit on the interactions between mothers and their children with complex care needs, especially their involvement, mutual attentiveness, and activity frequency and variation. The analysis yielded four main themes and eleven subthemes, see Table 3.

**Table 3.** Main themes, sub-themes and descriptions of the themes (N = 33).

Main theme	Subtheme	Description
Activities	Repetitive	The child repeats activities continuously, such as manipulating specific objects, swaying a piece of rope, et cetera. The activity appears to be a go-to for self-stimulation or self-calming.
	Individual	An activity that the child undertakes independently: playing or watching/listening to something.
	Together	Activities in which mother and child are engaged; leisure activities, such as playing a game or reading a book, or daily care activities, such as eating or bathing. This subtheme is mainly about shared attention during the activity.
Communication by the child	Positive affect	The child outwardly displays his/her feelings, emotions or mood, for example: laughing, crying, or turning away from an object. This subtheme includes focusing attention and seeking proximity to the mother (watching intently, moving closer, etc.).
	Taking the lead in interactions	The child shows initiative, without a prompt from the mother, such as grabbing an object by him/herself or initiating interaction with the mother by sound, gesture, or by physically approaching her.
	Responding to mother	The child’s response to the mother (active or passive) is when the mother initiates interaction or takes some other initiative towards the child, for example, by answering a direct question, following directions, or avoiding or ignoring.
Communication by the mother	Initiative towards child	The mother initiates interaction or takes some other initiative towards the child. For example, the mother talks about what is happening, asks questions, or initiates an activity with the child.
	Responding to child	The mother (actively or passively) responds to something the child does. For example, mirroring behaviour, verbal responses, encouraging or correcting.
	Talking about child/mentalising	The mother talks to the field researcher about the child’s characteristics, explains what is happening inside the child’s mind or tries to give meaning to their behaviour. This also includes sharing personal experiences concerning the child (experiences/struggles in day-to-day care, things they enjoy about their child).
About the ICT-Toolkit	Reflections on the use of the products	The mothers reflected on the use of a product from the ICT-Toolkit.
	General reflections	The mothers commented on the ICT-Toolkit in general (content, composition) and the study.

### Activities

#### Repetitive activities

*Baseline.* Andy, Brian and Dana appeared to have a default activity they engaged in when left alone. Andy often engaged in repetitive fidgeting with a shoestring. Brian regularly turned objects over and over for long periods and often threw small objects within his reach before crawling towards them again; this sequence was repeated. He also asked his parents to repeat the same songs continually. Dana’s favourite repetitive activity was finding toys that make an electronic sound, preferably loud, and holding them close to her ear. This behaviour could continue for hours (e.g., during a long car drive) if no one intervened. For Colin, no specific repetitive behaviour was mentioned.

*Intervention phase.* The repetitive activities mentioned in the baseline section above remained during the intervention phase. However, for Andy and Brian, they appeared to be less dominant and even could be circumvented or transformed by an ICT-Toolkit element. For example, Andy could be distracted from fidgeting by the switch button on several occasions. Rather than asking his mother to sing for him or throwing things, Brian remained engaged with the speaker for about half an hour.

#### Individual activities

*Baseline.* Andy, Dana and Brian’s activities included watching video clips on the television or tablet and manipulating objects. Additionally, watching video clips was also used to assist Brian with eating. Colin, who has limited motor functions, only engaged in solo activities related to physical activity, for example, using an adapted bike. When alone, he was usually in his bed box in the kitchen with the radio tuned on as a buffer against unexpected sounds that otherwise would easily trigger his epilepsy.

Brian and Dana regularly requested time alone in their room – Brian in his rocking boat, Dana in her closed bed box – by looking or moving towards it. Parents would help them settle and leave the room for about half an hour. The children would listen to music or engage with toys. Sometimes, Brian would also repeatedly open and close his bedroom door.

*Intervention phase.* During the intervention phase, the individual activities were still performed. However, several items from the ICT-Toolkit were added to the children’s repertoire. Usually, when their child was playing with a product, their mother closely observed, facilitated or encouraged them to play.

**Figure 1.**
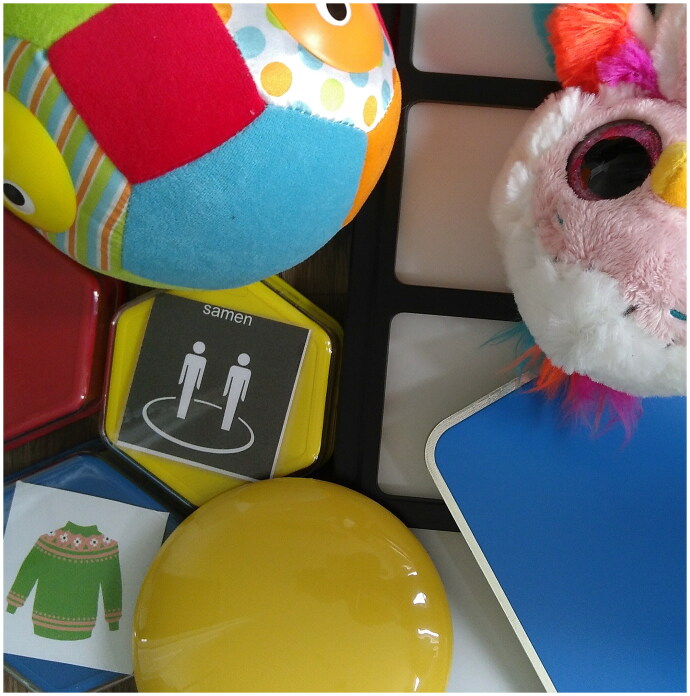
Examples of technological products suitable for their children in the ICT-Toolkit.

After some practice, Andy operated three speech buttons independently, with pre-recordings of his family’s voices and a song. Andy also often sought out the talking parrot (see Figure 1). According to his mother, Andy showed turn-taking behaviour towards the parrot: “*As if he has developed a new language. […] Andy made a sound and waited for the parrot to repeat the sound. He made all kinds of noises that way” (6:27).* His prolonged attention span when conversing with the parrot and the fact that it could distract him from fidgeting with the string were also remarkable (reported three times during the invention phase). Like Andy, Colin was also attracted to the parrot. His mother placed it near him and noted that Colin looked at it.

Since Brian’s first reaction to any (small) object in front of him is throwing it away, he enjoyed playing with the ball, but it did not seem to interest him more than other toys. Nevertheless, he was gentle with the vibrating speaker. This behaviour stood out as an unexpected success for his mother. *“Brian was involved with the speaker for half an hour, very much at ease. My husband and I find it remarkable that he does not throw it. Now and then, he touches it softly with his lips or fingers or puts his forehead against it”* (14:2).

Dana was interested in the items that provide sensory stimuli. In particular, the police car was used this way, with the sound off. *“Dana holds the police car against her mouth and nose. Her eyes were very close to the flashing lights. On and on, she pressed the yellow button for about 3 s before releasing it briefly and pressing again.” (37:2).* Similarly, on one occasion, the vibrating speaker held her attention remarkably long when the speaker was attached to a chair, and she put her teeth on the chair’s seat for over thirty minutes.

#### Activities together

*Baseline.* Different activities were provided by Andy, Brian, and Colin’s mothers to engage them in activities: Andy’s mother offered different stimuli and activities; an important goal for her was to keep Andy physically active by going on walks or dancing together. In general, she was in control of choosing the activity and the duration; Brain’s mother primarily engaged with him by singing songs. He often initiated this by requesting songs *via* manual signs or saying some of the words. Other shared activities included looking at pictures together, where both pointed at pictures in the book; Colin and his mother were engaged in many activities together, consisting of physical therapy, everyday caregiving moments, or just being around each other. For example, Colin was often held on his parents’ lap when fed. Besides, he spent most of his time at home in a bed box in the kitchen, where he was included in family life.

Dana and her mother were less inclined to undertake reciprocal activities, even when in each other’s proximity. Dana’s mother mostly silently observed Dana’s playing or repetitive behaviour. For Dana’s mother, it was counter-intuitive and hard to accept that Dana preferred to spend most daily activities alone, without (verbal) interaction. However, she recently learned that Dana enjoyed being stroked gently with an object in silence (making the interaction less stimulating).

*Intervention phase.* During the intervention phase, the number of activities undertaken increased in most dyads; the mothers experimented with different toys and devices with their children.

Andy used the power switch (see Figure 1) more than any other product, prompting more diversity in shared activities. Andy and his mother used it several times together in the kitchen to prepare a dessert. Moreover, two instances were reported in which Andy would stop fiddling with his favourite string when his mother showed the power switch. Reportedly, this successful distraction from fiddling had rarely happened before.

Brian responded most notably to the vibrating speaker, which took some of the pressure off his mother to sing the same songs repeatedly. Instead, during the sixth observation, Brian’s interest and sustained attention prompted the rest of the family to listen to the speaker together. *This is the first time I have seen them all together. Before, I usually saw Brian with his mum and [his brother] with their father. Now the four of them are gathered around the speaker. They are all smiling.* (14:13)

Dana’s mother continued to observe Dana when she was playing silently, and Dana continued to engage in solitary play for the majority of the time. However, Dana’s mother mentioned: *“…This police car toy helps in the sense that… it was something new, so Dana also did new things that I had never seen before. By watching really closely, you can see that thereby she has made a step in her development. I become more aware of that.”* (25:19)

### Communication by the child

#### Positive affect

*Baseline.* Most of the children showed positive affect non-verbally. For instance, Andy showed positive affect by laughing, making loud noises, and bouncing up and down. Brian displayed positive affect by smiling and laughing. Colin and Dana, on the other hand, did not often initiate positive affect but sometimes reacted to their mother’s behaviour by smiling and sometimes showed appreciation in response to nurturing and caring efforts by cuddling. Other forms of positive affect were shown non-verbally by Andy, grinding his teeth and moaning, and Brian crying or wailing.

*Intervention phase.* No changes in positive affect towards their mothers were observed. Conversely, except for Colin, more positive affect was observed when they were engaged with the ICT-Toolkit items. More smiling (enjoyment) was reported, e.g., Brian and Dana smiled a lot while using the vibrating speaker and the story sequencer with a recording of his mother’s voice. Andy regularly smiled and repeatedly imitated the motions of the parrot (by shrugging). On a few occasions, Andy also displayed disinterest by walking away from his mother or ignoring her when she offered him an item.

#### Taking the lead in interactions

*Baseline.* The mothers of Andy, Brian and Dana took the lead by promoting or facilitating their child’s action or activity. There were times when the children took the initiative to express their needs, e.g., being moved or other basic needs such as wanting to bathe or listen to music (Andy, Brian, Dana), and Brian took his mother’s arm to make her grab the object he wanted. Brian initiated activities such as opening/closing doors, watching TV, reading books, throwing objects, and going outside or to his room. Andy and Dana did not take as much lead in different activities but instead focused on their favourite object, going outside or cuddling. As for Colin, he did not take observable initiative.

*Intervention phase.* No data is available for Colin regarding his taking the lead or communicating non-verbally. More initiatives were reported by Andy, who regularly took the parrot and the ball to play; Brian appeared to be less reliant on his mother to entertain him, as he played frequently and was at ease with the speaker. Dana took several initiatives by touching the speaker (when her mother had turned it on) and the police car and playing with the switch button and the ball. Towards their mothers, there were more initiatives for communication, such as Andy tended to look at his mother if he appreciated an item or activity (interview with mother). During one interaction, he asked explicitly (by gesturing) to hear the recording by moving the yellow switch button. Brian asked explicitly (gesturing) for the yellow switch button to hear the recording.

#### Responding to their mother

*Baseline.* The children responded to their mothers by turning their heads towards the source of the sound and sometimes by smiling (Colin), by showing facial expressions, pointing, grabbing, saying “yes”/“no” or giving other one-word responses (Brian) or choosing between two options *via* pictures on a tablet computer by accepting or pushing away objects (Dana). Andy seemed to understand what his mother requested and responded to her most of the time by either allowing, cooperating or rejecting her request. Nevertheless, at times it was difficult for their mothers to know whether they did notice the initiatives their mothers took and/or understood their requests.

*Intervention phase.* In all four dyads, there appeared to be more turn-taking interactions between child and mother in trying various ICT-Toolkit items. On several occasions, the mother demonstrated using an item before handing it to their child, to which the child responded cooperatively or not. No differences in responding to the mother stood out.

### Communication by the mother

#### Initiatives towards child

*Baseline.* All four mothers exhibited non-verbal and verbal communication in their attempts to initiate contact. The mothers communicated *non-verbally via* physical proximity (e.g., sitting beside their child), cuddling, nurturing and restricting or directing (e.g., taking their child to another place). Additionally, all four mothers often communicated v*erbally* towards their child by describing the situation, interpreting what the child was doing, announcing what would happen, and telling the child what (not) to do (see also 3.4). Most observed mother-child *interactions* were started by the mother proposing a new *activity*.

For non-verbal communication, referral objects were used (e.g., Andy: shoes for "going outside") or supportive signs or pictures (Dana). During verbal communication, mothers regularly gave their child a choice of two (types of drinks, games) by showing them the two options and asking which one they preferred (Andy and Dana). Mothers would also talk and sing (Brian and Colin). Colin’s mother spoke a lot as she wanted to keep him alert to prevent him from being startled by sudden sounds, which would trigger his epilepsy. During bodily contact with Colin (i.e., holding him onto her lap), moments of (pleasant) shared attention while feeding him and frequently exchanging smiles were observed. When Andy kept resisting or ignoring his mother’s suggestions, she eventually insisted (e.g., taking the shoestring out of his hand or putting his jacket on).

*Intervention phase.* In general, the mothers appeared to apply the same communicative style and strategies when introducing items from the ICT-Toolkit, but more Dana’s mother showed more non-verbal communication. Also, on some occasions, the mothers physically and verbally demonstrated a Toolkit activity to motivate their children. Colin’s mother placed the parrot in his vicinity when she made dinner. By imitating his sounds or something she would say to him, the parrot kept Colin alert – thereby preventing him from sudden scares in response to the unexpected clanging of pots and kitchen tools, typically triggering a seizure. During the interview, she explained that this interaction and tool allowed her to remain engaged with her child (31:2).

#### Responding to child

*Baseline.* All the mothers used verbal and non-verbal communication when responding to their children’s attempts to communicate or showed clear communication. Their responses sometimes focused on attempts to prevent undesired behaviour like setting boundaries throwing and breaking objects (Brian), or responding affirmatively to repetitive or unsafe behaviours like when Andy put things in his mouth or lashed out at his mother. Nevertheless, mothers often found it challenging to respond: Andy’s mother found it difficult to distinguish and understand Andy’s various noises, and Dana’s mother often did not get clear responses from Dana. A response of enjoyment was observed during caring moments, such as Colin’s mother smiling back at him while feeding him on her lap, and Dana’s mother mentioned that the firm cuddle Dana occasionally gave her during bathing, nurturing or dressing meant a lot to her since these were the only explicit expressions of relational appreciation she got from her daughter.

*Intervention phase.* Except for Colin, throughout the intervention phase, and contrasting with the baseline observations, the mothers followed their children more in their play. For example, Andy’s mother supported Andy’s behaviour when making dessert using the power switch but stopped when Andy walked away. Brian’s mother could sit back and watch Brian engage with a toy, playing independently; Dana’s mother consciously and silently watched how Dana touched the new toys. Furthermore, Andy’s mother kept trying to get him involved (e.g., blowing leaves in a basket with the hairdryer attached to the power switch and Dana’s mother, although she reported that this was feeling counter-intuitively to her, she engaged more in the silent and tactile interactions which Dana seemed to appreciate, i.e., by touching her legs and arms with small objects or toys.

#### Talking about the child/mentalising

*Baseline phase.* Besides directly interacting with their child, the mothers also talked to the observer about their children. They often explained and interpreted their children’s behaviours, conditions, activities and interactions and named things or activities their children enjoyed.

The mothers attempted to explain any atypical social behaviours of their children. For example, most mothers mentioned that their child does not distinguish between people, is slow in recognising specific people besides their family and significant others, and does not understand the concept of saying goodbye. Mothers shared their medical and developmental/psychological analyses of their child’s behaviour: and the effects of it on their day-to-day life and caregiving: E.g., Dana’s mother mentioned that she and Dana’s father tended to ask too much of Dana which could contribute to Dana being overstimulated but became more cautious and patient when trying to teach Dana a new communication method.

None of the mothers was sure about their child’s sensory perception; they struggled to understand what their child could see or hear and/or the link between these children’s developmental level and their short attention span.

They also discussed the ongoing challenge of finding new, fitting, sustainable activities and interactions with their child. Furthermore, in the same vein, they found it challenging to find toys that suited their child’s needs and interests.

*Intervention phase.* Also, during the intervention phase, similar themes mentioned above were identified. The primary reported difference was that mothers talked about the perceived new possibilities in communicating with their children by applying ICT-Toolkit items. The most striking examples were when Andy’s, Brian’s and Dana’s mothers were surprised by their child’s remarkably long attention span when they were engaged with their favourite item. While observing their child, they talked about how their child used the ICT-Toolkit items and which features could and could not attract them to the items.

### About the ICT-Toolkit

#### Reflections on the use of the products

None of the mothers spent much time reading the (digital) information package of the ICT-Toolkit as they preferred to spend their (limited) time and energy testing the item intuitively by just trying and doing it. Also, they did not pay much attention to the ICT-Toolkit user manual with suggestions for daily use. However, the ICT-Toolkit item manuals’ online availability and user suggestions were appreciated.

During the intervention phase, the children showed prolonged sustained attention when engaging with their favourite ICT-Toolkit item. The most popular products were the microswitch, police car, talking tiles, talkback parrot and vibrating speaker (Table 4). The strong stimuli emitted by the police car made it appealing to two of the children. However, for the same reason, their mothers expressed concerns about the police car potentially triggering epilepsy. The mothers used items based on the reaction of the child. For example, Brian disapproved of the talking parrot (which he was afraid of) and the yellow button was not applied (which Brian and Dana did not seem to understand), but Colin did engage with the talking parrot.

**Table 4.** Usability of the products in the ICT-Toolkit for each participant.

Product	Andy	Brian	Colin	Dana
Microswitch	++	–	+	+
Power switch	+	na	na	–
Police car	++	+	na	++
Talking tiles	+	+	na	na
Parrot	++	–	+	–
Vibrating speaker	na	++	++	++
Computer interface	–	na	+	na
Interactive ball	+	+	na	+

++ Successful use of the product; + use of the project; – not successful use of the product/was not used; na Not applicable/no data about positive or negative experiences.

The PAL-pad was used the least. None of the mothers had recognised its applicability as a micro switch, even though the use was briefly explained during each ICT-Toolkit drop-off. This was also the case for the story sequencer. Although Brian’s mother had tried it out briefly to record songs on it, she decided that its recording ability was too short to interest Brian. The assistive software and eye tracker were also not used as it was deemed unsuitable, or mothers experienced the installation process and use too time-consuming and challenging (Brian, Andy and Colin).

All mothers suggested that outdoor toys would be a valuable addition to the ICT-Toolkit, since, with the current content, its use was limited to indoor use. Secondly, they proposed adding items stimulating exercise and/or tactile activities and interaction. Thirdly, they asked for more suggestions for relevant assistive technology applications.

#### General reflections

All the mothers mentioned that they found the assistive technology highly beneficial. The use of the ICT-Toolkit opened up new possibilities (e.g., the use of the switch button for Andy), it supported “out-of-the-box thinking” on the use of assistive technology (noted by Brian’s mother), it inspired to apply computerised activities (e.g., to keep Colin alert and to familiarise him with new sounds or ease him into unexpected ones) and reminded parents to observe and follow the child before wanting to take the initiative or action (e.g., Dana’s mother became more aware of this). Although the observation home visits did contribute to becoming more aware of their behaviour (e.g., mentioned by Dana’s mother), it was also experienced as intensive and some lack of clarity on the goals of the observation sessions (e.g., Dana’s mother expected that the observations would be solely focused on the use of the Toolkit). All the mothers appreciated their participation. *It was really nice to participate. To do so was already out of my comfort zone; in general, I would not take part in things like this. But I am glad that I did partake this time. I really enjoyed being involved in nice things for a while. Not in caring or medication, stuff like that. Just something fun. I truly liked it.* (reported by Brian’s mother, interview). Some parents decided to purchase some items (e.g., the switch button for Andy and the vibrating speaker for Brian). Still, for some families, these items also appeared to be expensive. Only Colin’s mother downloaded software programs, as suggested in the ICT-Toolkit, to be used with the computer interface and power switch to provide extra sensory stimulation for Colin. It was, however, also noted that parents are limited in what they can offer their children with complex care needs regarding suitable interaction and activities (mentioned by Dana’s mother).

## Discussion

This paper examines how dyad interactions were affected when the ICT-Toolkit was easily available. Specifically, the study explored the effect of the availability of the ICT-Toolkit, focusing on their involvement, mutual attentiveness, and activity frequency and variation. The first aim was to explore how the availability of the ICT Toolkit influenced the interaction between parent and child in terms of the activities they undertook. The findings indicate that, although repetitive activities by the children were still present during the intervention phase, some repetitive behaviour appeared to be less dominant and could be circumvented or even transformed by an ICT-Toolkit element. In addition, some new individual activities appeared during the intervention phase. Several Toolkit items became part of the children’s repertoire. The secondary aim was to gain insight into which elements of the ICT-Toolkit were involved in those interactions and activities. It was observed that children showed more the lead in their behaviour than before, although no changes in affect towards the mothers were observed. Concerning the third aim, all children showed more attention when using specific Toolkit-items, most notably the vibrating speaker, talking tiles, police car and parrot.

Three out of four dyads showed a noticeable increase in activities between mother and child, including more turn-taking interactions. All mothers appeared to apply the same communicative style and strategies during the baseline and intervention phases. However, contrasting to the baseline observations, three mothers engaged in a more observational and less verbal way of interacting with their child, following the child’s lead, when using ICT-Toolkit items. This approach of “following the child’s lead” is in line with other types of interventions for children with complex care needs [35–37] that focus on stimulating the development of a secure attachment relationship through sensitive and responsive parenting, and the way the ICT-Toolkit was used and studied may also contribute to this. When following the child’s lead, the parent will be better able to adapt communication strategies to the abilities and preferences of the person with complex care needs. Through improved attunement, the interaction becomes more interactive, engaging, and, therefore, more meaningful.

The challenges of using technology mentioned by authors like (28] were confirmed at the outset of the current study as, due to time restraints [38], mothers reported that they did not have and take enough time to gain knowledge or experience in using assistive technology. Also, although all mothers had been looking for new, stimulating activities for their children, they struggled to find information about available products. Furthermore, the high costs for tailor-made technology for “children with disabilities” was also mentioned as a barrier. Therefore, having the ICT-Toolkit at home did stimulate mothers to explore new activities and try out the products while they were available. They were all surprised by the impact of at least one item on their child, most notably their child’s increased attention span for that particular item. Furthermore, and in line with the *theory of attunement* [39], our findings suggest that adding new, appropriate stimuli to a situation prompted new actions and engagement from some of the mothers, thereby alluding to the suggestion that attunement is not only a matter of personality and interpersonal communication but also of materiality [39].

During the intervention phase, compared to the baseline phase, there was more individual attention of the child in three out of four dyads, providing their mother with a little more (highly appreciated) private time. Said prolonged individual attention of the child sometimes entailed new behaviour, to the mother’s surprise: e.g., turn-taking with the parrot (Andy), and not throwing but gently attending to the speaker and talking tiles (Brian). However, new prolonged attention towards an item was sometimes met with ambivalence by the mother: when her child seemed to enjoy the speaker’s vibrations on the jawbone, she wondered how that would help her develop (Dana). Also, in some dyads, there was a fear of negative effects, such as that the lights of the police car could trigger epileptic seizures when the child played alone. Only for Colin, the ICT-Toolkit did not facilitate an increase in shared activities, but surprisingly due to the experience with the “talking parrot”, his mother took more initiative to find sounds and music that would keep Colin alert and to familiarise him with new sounds or ease him into unexpected one which could trigger the seizures. Also, his mother mentioned that, for Colin, the intervention phase was still too short, and in practice, it is advisable to provide the ICT-Toolkit for a longer period when needed.

In terms of communication and interaction, all mothers noticed, to some extent, their child’s “new” behaviour when interacting with an ICT-Toolkit item (and/or the research setting). In three out of four dyads, the mothers also appeared more sensitive and responsive to the communicative signals of their children when they were trying new items together. Some of the ICT-Toolkit items were part of small, at least temporary, changes in the dynamics between mothers and their children with complex care needs. For instance, for Brian and Colin, the vibrating speaker and the parrot enabled more independence for both mother and child, and for Andy, the microswitch facilitated an unprecedented quality time between mother and child in the kitchen.

Lastly, all mothers applied *close observation* when interacting with their children through ICT-Toolkit items. Their child’s reported newly observed behaviour appears to be connected to the focused attention of the mothers. However, it remained unclear whether this was a direct result of the latter’s sharpened attention (i.e., they observe differently, so they see different things) or an indirect consequence (i.e., they are more attentive to their child, who acts differently). Despite this this lack of clarity, these findings resonate with the *theory of attunement* [39], which suggests that adding a stimulus might stimulate new ideas and elicit new actions. In that respect, most remarkably, Andy, Brian and Dana’s mothers, when interacting with the ICT-Toolkit items, could more easily let their children explore and attune to their preferences. Again, the finding of Andy, Brian and Dana’s increased attention span – to their mothers surprisingly long – is relevant here. None of the mothers claimed to be certain about their child’s sensory perception; they all struggled to understand what their child could and could not see or hear. This study’s findings imply that the technological items provided a different quality to their play than other items already in the house.

Contrary to our intentions, mothers hardly used the ICT-Toolkit instructions and information booklet (also when it was digitally provided in phase 2) or the instruction videos. They reported not having/experiencing enough time, energy, or “head space” to focus on the instruction, being in their survival mode. Getting hold of all the instructions and information and adequately exploring the toolkit’s possibilities with their everyday hectic was perceived as too much. This challenge highlights the ambiguity parents experience when using technology – they express the need but do not really take the time to read about it, and then are positively surprised when used and results are seen.

## About the design

Interpreting these results must be done cautiously since some of the effects can also be connected to the study design, which entailed a field researcher as a participant-observant in family homes for a considerable time. The main advantage of this method was that it allowed an in-depth understanding of the individual situation of the participants. This advantage is especially relevant because of the large variability in the needs and abilities of children with complex care needs. The parents of children with complex care needs possess tangible knowledge that can best be transferred daily [40]. The field researcher can report all relevant information in a particular setting and is not restricted by standardised protocols. Furthermore, to prevent missing out on information, observation reports and interviews were validated by the mothers.

However, a consequence of this participatory approach is that the field researcher affects what happens during the sessions. A clear example is that several mothers in the intervention phase would start demonstrating their use of the ICT-Toolkit items during the observation sessions. These demonstrations might have led to an overrepresentation of the use of technology in our observation reports compared to the typical home situation, although the mothers also described using the products at times when the field researcher was not around. Furthermore, the mere presence of the field researcher may have stimulated the mothers to be more patient or persisting (i.e., due to social pressure) and the child to be more withdrawn, disinterested or distracted (i.e., due to the presence of a non-significant other/stranger) when trying a new product than they would usually be. For example, Dana’s mother noticed that the field researcher was sitting and watching her daughter rather than talking to and engaging with her. In the evaluative interview, she reflected that this attitude changed her behaviour towards her child: *"I saw him make those observations. So, at one point, I also decided just to sit down and look at what Dana was actually doing. This toy helps in the sense that… it was something new, so she also did new things that I had never seen before. By really looking closely, you can also see that she has made a step in her development or something. You become more aware of it."*

Beforehand, the research team had been reluctant to let the field researcher take the lead in using the ICT-Toolkit items, eager to leave as much as possible up to the mother and child. However, in some instances, it would happen that the field researcher suggested new uses, would assist in learning how to operate a device, or would actively refer parents to the available instructions. This was particularly so when he perceived that mothers did not read the instructions or watch the videos for product use, and their preferred “learning by doing” was unsuccessful, or when they repeatedly asked him for advice. On the few moments that some mothers appeared to give in or refrain from starting when they had not read or watched the ICT-Toolkit instructions – due to a lack of energy, attention, time and patience in their everyday hectic – it proved to be helpful that someone (i.e., the field researcher) took initiative to try out with, besides and/or for them.

## Implications for practice

Our results imply that bringing assistive technology to the homes of children with complex care needs can be fruitful: mothers were triggered to try out several ICT-Toolkit products that they had not used before, which proved promising to some extent. At the same time, since their experimenting was limited to specific applications of specific items, they did not utilise the full potential of the ICT-Toolkit. As a result, the question is whether more extensive use would be preferable, and if so, how to facilitate that.

In line with (28], we conclude that stimulating an increase in knowledge and experience helps to facilitate a more open attitude towards using assistive technology from mothers. In our study, this open attitude of the mother appears to be intertwined with her experiencing success and – to some extent – being able to collude with her partner and/or the field researcher. After all, without exception, the mothers kept saying that their family life was very busy, they were very tired, and they (therefore) were inclined to stick to their daily routines and patterns. Our study illustrates that adding technology-supported products to these family lives may contribute to attunement and independent and interactive play. Specifically, this assistive technology is supportive because of the predictability, uncomplicated and highly perceivable stimuli it provides with minimal effort; there is a maximum effect. Nevertheless, the products must be combined with clear, tailored instructions and helpful support for parents. Regarding the latter, to have a support worker playing and moving along in everyday family life where the assistive technology is introduced (as the field researcher did). For the same reason, and also to reduce time investment for the families, regular assessments of visual and auditory impairments are needed in the home environment.

Since we saw that mothers did not engage with the instruction manual and videos, support to use the full potential of the ICT-Toolkit seems pivotal. In line with [40], we suggest combining this support with listening closely to the parents’ perspectives on the interests and possibilities of their child. At the same time, we see some evidence of the potential to encourage parents to step out of their comfort zone and engage in new and unthought-of activities (e.g., Brian was attracted to the vibrating speaker and did not throw\it, contrary to his mother’s expectations; several children showing a longer attention span to items from the ICT-Toolkit products than other toys). In general, engaging in new activities – whether assisted with technology or not – may help to get to know new sides of a child and thus help widen the perspectives of its parents on meaningful interactions and activities [10].

## Future research

The present study specifically focused on mother-child dyads and how technology would influence the dynamics between these two concerning their involvement, mutual attentiveness, and activity frequency and variation. However, in everyday life, these dyads functioned within a wider context of family members and care professionals. Everyone involved had their preferences, needs, and perspective on the child’s needs. In our research, the role of fathers remained unaccounted for since they appeared to work, whereas the mothers were mostly at home with their child(ren). It would be worthwhile to investigate how our findings relate to meaningful activities and interactions between fathers and their children and how assistive technology might benefit them. Aligned with this, it would also be intriguing to explore how the ICT-Toolkit could impact interactions between siblings and/or peers.

In addition, the field researcher in our study aimed to participate in daily activities whilst causing minimal disruption. However, his presence did stimulate the dyads to use the ICT-Toolkit during his visits. After the intervention period, the items were forwarded to the next participants. Nevertheless, some parents mentioned that the intervention time was too short and others that they now knew which kind of material they wanted to purchase. Future studies could also focus on how these products, in the long term, may be integrated into daily life and the effect thereof.

Lastly, our findings might inspire studies into the potential of assistive technology in other age groups, for example, older persons with complex care needs [41], in both professional and residential settings.

## Conclusions

The primary aim of our study was to gain insight into whether the introduction of the ICT-Toolkit would influence the involvement, interactions and participation of mothers and their children with complex care needs – through participant observation. We conclude this was the case since several items in the Toolkit facilitated the participating mother-child dyads to explore new activities and interactions. Furthermore, in some instances, using the ICT-Toolkit items led mothers to mentalise about the needs of their child and how their behaviour may influence the behaviour of their child. Generally, compared to the baseline phase, during the intervention phase, mothers looked at their child differently, and/or enabled the child to explore by following the child’s lead and supporting the child’s initiatives or expand its attention span.

Our secondary aim was to map which items/elements in the ICT-Toolkit were instrumental in this effect. At first, striking a clear conclusion is harder here since some of the positive effects may have been facilitated or mediated by the presence of the field researcher. The conclusion is that a fruitful introduction of assistive technology within family homes might largely depend on an external party introducing the ICT-Toolkit products and assisting and observing in the try-out phase. After all, although the user’s manual did provide some guidance to some of our participants, the mere provision of some technological items and a user’s manual appeared insufficient to stimulate them to get started. Tailored and hands-on assistance in (hectic) home settings can contribute to applying the full potential of assistive technology or yield more insight into options that would make specific items more suitable for specific children and their families.

## Data Availability

Data are available on request from the corresponding author.
